# Expression Profile of CD157 Reveals Functional Heterogeneity of Capillaries in Human Dermal Skin

**DOI:** 10.3390/biomedicines10030676

**Published:** 2022-03-15

**Authors:** Katarzyna Michalak-Micka, Dominic Rütsche, Lukas Johner, Ueli Moehrlen, Thomas Biedermann, Agnes S. Klar

**Affiliations:** 1 Tissue Biology Research Unit, Department of Surgery, University Children’s Hospital Zurich, 8032 Zurich, Switzerland; katarzyna.micka@kispi.uzh.ch (K.M.-M.); dominic.ruetsche@kispi.uzh.ch (D.R.); lukas.johner@gmail.com (L.J.); ueli.moehrlen@kispi.uzh.ch (U.M.); thomas.biedermann@kispi.uzh.ch (T.B.); 2 Children’s Research Center, University Children’s Hospital Zurich, 8032 Zurich, Switzerland; 3 University of Zurich, 8006 Zurich, Switzerland; 4 Department of Surgery, University Children’s Hospital Zurich, 8032 Zurich, Switzerland

**Keywords:** CD157, CD157 receptor, immune cell adhesion, vascular network formation, blood capillaries, lymphatic capillaries, angiogenesis, myeloid cells, microvascular endothelial cells, skin bio-engineering

## Abstract

CD157 acts as a receptor, regulating leukocyte trafficking and the binding of extracellular matrix components. However, the expression pattern and the role of CD157 in human blood (BEC) and the lymphatic endothelial cells (LEC) of human dermal microvascular cells (HDMEC), remain elusive. We demonstrated constitutive expression of CD157 on BEC and LEC, in fetal and juvenile/adult skin, in situ, as well as in isolated HDMEC. Interestingly, CD157 epitopes were mostly localized on BEC, co-expressing high levels of CD31 (CD31^High^), as compared to CD31^Low^ BEC, whereas the podoplanin expression level on LEC did not affect CD157. Cultured HDMEC exhibited significantly higher numbers of CD157-positive LEC, as compared to BEC. Interestingly, separated CD157^−^ and CD157^+^ HDMEC demonstrated no significant differences in clonal expansion in vitro, but they showed distinct expression levels of cell adhesion molecules, before and after cytokine stimulation in vitro. In particular, we proved the enhanced and specific adherence of CD11b-expressing human blood myeloid cells to CD157^+^ HDMEC fraction, using an in vitro immune-binding assay. Indeed, CD157 was also involved in chemotaxis and adhesion of CD11b/c monocytes/neutrophils in prevascularized dermo–epidermal skin substitutes (vascDESS) in vivo. Thus, our data attribute specific roles to endothelial CD157, in the regulation of innate immunity during inflammation.

## 1. Introduction

Human CD157, also known as bone marrow stromal antigen 1 (BST1), has recently gained increased interest, as an important immune cell adhesion molecule [1]. CD157 is a glycosyl phosphatidylinositol-anchored membrane protein that belongs to the CD38 family [1]. Despite being a NAD^+^-metabolizing ectoenzyme role, the receptor role of CD157 has been clearly delineated with the identification of its high affinity binding to ECM proteins, such as fibronectin and transduction of intracellular signals [2,3].

Human CD157 is mainly expressed by human blood myeloid cells, but also by other cells, including the vascular endothelium and/or tissue-resident vascular endothelial stem cells, as well as synovial and follicular dendritic cells [2,4,5,6]. In particular, CD157 has been found in vascular endothelial junctions, where it regulates the transendothelial migration of myeloid cells [4], by controlling the interaction with β1 and β2 integrins [7]. In particular, αMβ2 integrin (CD11b; MAC-1) is a marker, highly expressed on myeloid populations, such as monocytes/macrophages and granulocytes, but also on some dendritic cells [8]. 

Previous studies revealed that CD11b is a receptor for complement, fibrinogen, and endothelial cell adhesion molecules, including intercellular cell adhesion molecules-1/2 (ICAM1/2). Thus, CD11b has been shown to mediate myeloid cell adhesion, migration, chemotaxis and immune cell accumulation during inflammation [9,10,11]. Importantly, Funaro et al. showed that CD11b acts as a receptor for CD157 to regulate myeloid cell adhesion and migration [12], and plays an important role during inflammation, where it regulates the adhesion and trans-endothelial migration of neutrophils and monocytes [7,12].

CD157 was shown to regulate the binding to ECM components, such as fibronectin over integrin α_5_β1/CD29 and integrin αvβ1/CD51 [3]. For example, HUVECs displayed a substantial amount of α5β1 receptors at the cell surface, in monolayer in vitro cultures [13]. Further, previous reports demonstrated that EC, showing increased mitotic index in vitro, correlated closely with increased α_5_β_1_/CD29 integrin expression [14]. Further, the α_5_β_1_ integrin was shown to be highly expressed in quiescent EC, whereas other integrins are specifically expressed during the process of angiogenesis [15].

Furthermore, it has been shown that CD157 signaling activates the SRC-family protein, tyrosine kinase, phosphoinositide 3-kinases (PI3Ks)/protein kinase B/Akt, mitogen-activated protein kinase (MAPK) and extracellular signal-regulated kinase (ERK) pathways and, thus, increased survival of acute myeloid leukemia cells [16]. Further, CD157 directly activates SRC/ERK and PI3K/AKT signaling pathways in human endothelial cells. Those signaling pathways play a crucial role in the development of both blood [17] and lymphatic endothelial cells [18]. In particular, endothelial ERK signaling controls lymphatic fate specification during embryonal development [18]. However, the CD157 regulation of SRC/ERK and PI3K/AKT signaling pathways in human primary endothelial cells has not been investigated so far.

Importantly, previous investigations demonstrated that CD157^+^ endothelial cells (EC), derived from mouse liver, show enhanced clonal expansion, proliferation, and blood vessel formation, under physiological conditions after transplantation [5,19]. Thus, Wakabayashi et al. concluded that CD157-positive ECs’ fraction in mice represent tissue-resident vascular EC stem cell population [5]. However, still, little is known about the specific function of human CD157 molecules on distinct human dermal microvascular endothelial cell (HDMEC) subpopulations, including blood endothelial cells (BEC) and lymphatic endothelial cells (LEC). 

Herein, we report, for the first time, the specific expression pattern of CD157 on juvenile/adult (j/a) and fetal (f) skin HDMEC, as well as in bio-engineered prevascularized skin substitutes (vascDESS) in vivo. Moreover, CD157^+^ and CD157^−^ HDMEC were characterized with respect to their relative clonogenic and proliferative potential, as well as their inflammatory response, following cytokine treatment. Our analysis further revealed some unique immune cell-binding features for human HDMEC, positive for CD157, when compared to CD157 negative cells. Thus, our data imply heterogeneity among dermal EC subtypes, including diverse metabolic and immune functions. 

## 2. Materials and Methods

### 2.1. Cell Isolation and Culture

All experiments were performed according to the Declaration of Helsinki Principles and after permission from the Ethics Commission of the canton of Zurich. Human skin-derived dermal microvascular endothelial cells (HDMECs), dermal fibroblasts (HDF), and keratinocytes (KC) were isolated and expanded from: fetal skin obtained from Spina Bifida operations between 24 and 26 week of gestational age (University Children’s Hospital Zurich, ethics approval: BASEC No. PB_2020-00066), juvenile/adult skin including foreskin, scalp, skin from the hands, abdomen, legs and arms from children ≤18 years (University Children’s Hospital Zurich, BASEC No. 2018-00269); from adult patients (18–65 years old) from various body areas such as breast and abdomen (Kantonsspital Aarau, ethics approval: BASEC No. 2018-00269) as previously described. HUVEC were purchased from ScienceCell (order no. 8000, Basel, Switzerland).

Blood-derived immune cells were collected from peripheral blood mononuclear cells (PBMCs) isolated from blood donated by healthy volunteers. Informed written consent was obtained from all blood donors at the Zurich Blood Donation Center (Zurich Blood Transfusion Service of the Swiss Red Cross, Schlieren, Switzerland, www.zhbsd.ch; accessed on 01.11.2021) according to the guidelines of the local ethics committee.

### 2.2. Cell Preparation for Flow Cytometric Analysis and Cell Sorting (FACS)

The phenotype of freshly isolated and cultured HDMEC (P1–P3) and/or BEC and LECs cells was determined by flow cytometry analysis. Cells (1 × 10^6^) were incubated for 30 min at 4 °C with primary antibodies: anti-human CD31-PE (clone WM59, 1:50, BD Bioscience, Allschwil, Switzerland), anti-human CD157-Alexa Fluor 488 (clone 534509, 1:50, R&D, UK), anti-human Podoplanin-Alexa Fluor 647 (clone: NC-08, 1:50, BioLegend, Lucerne, Switzerland), anti-human HLA-DR-Pacific Blue (clone: LN3, 1:50, BioLegend, Lucerne, Switzerland), anti-human CD54-Alexa Fluor 488 (ICAM1) (clone: HCD54, 1:50, BioLegend, Lucerne, Switzerland), anti-human CD102-PE (ICAM-2) (clone: CBR-IC2/2, 1:50, BioLegend, Lucerne, Switzerland), anti-human CD62P-APC/Cyanine7 (P-Selectin) (clone: AK4, 1:50, BioLegend, Lucerne, Switzerland), Zombie Aqua (live/dead dye) (1:600, BioLegend, Lucerne, Switzerland) or isotype-matched control antibodies and then washed twice with FACS buffer (0.5% human serum albumin, 0.5 mM EDTA in PBS). Following isotype controls were used at the concentration as specific antibodies: isotype control PE (clone MOPC-21, BD Pharmingen, Allschwil, Switzerland), isotype control Alexa Fluor 488 (clone 11411, Novus, UK), isotype control Pacific Blue (clone: MOPC-21, BioLegend, Lucerne, Switzerland), and isotype control Alexa Fluor 647 (clone MOPC-21, BioLegend, Lucerne, Switzerland). After incubation, cells were washed with FACS buffer and then analyzed using a BD LSRFortessa flow cytometer (BD Biosciences, Allschwil, Switzerland).

Hierarchical steps during gating strategy involved: (a) identification of the cell population of interest using forward versus side scatter (FSC vs. SSC) gating, (b) exclusion of dead cells using Zombie Aqua staining, (c) gating on CD31^+^ endothelial cells, and (d) discrimination between CD31^+^PDP^−^ BEC and CD31^+^PDP^+^ LEC cells (Supplementary Figure S1).

Accordingly, distinct BEC populations (CD31^+^PDP^−^CD157^+^ and CD31^+^PDP^−^CD157^−^) as well as distinct LEC cell populations (CD31^+^PDP^+^CD157^+^ and CD31^+^PDP^+^CD157^−^) were sorted using a BD FACSAria^TM^ III (BD Biosciences, Allschwil, Switzerland).

### 2.3. Immune Cell Binding Assay In Vitro

FACS-separated CD157^+/−^ BEC and LEC (P1) were seeded onto 24-well plates (Corning, Root, Switzerland) until reaching 80–90% confluency. Peripheral blood mononuclear cells (PBMCs) were isolated from fresh whole human blood using buffy coats (Zurich Blood Transfusion Service of the Swiss Red Cross, Schlieren, Switzerland). Briefly, blood was diluted with PBS (1:1) and gently layered over an equal volume of Ficoll-Paque PLUS (GE Healthcare, Opfikon, Switzerland) and then centrifuged for 30 min at 400 g without brake. After centrifugation, the PBMC fraction was washed twice with PBS followed by 30 min staining with anti-human CD11b-PE antibody (clone M1/70, 1:20, BD Pharmingen, Allschwil, Switzerland) at 4 °C in the dark. After washing with FACS buffer, CD11b-positive myeloid cells were immediately sorted using a FACS ARIA III 4L (BD Biosciences, Allschwil, Switzerland. In the next step, EC in 24-well plates were stained with CellTracker Deep Red Dye (Invitrogen, Zug, Switzerland) and sorted myeloid fraction was stained with CellTracker Red CMTPX Dye (Invitrogen, Zug, Switzerland) according to the manufacturer’s instructions. Stained cells were then incubated separately in incubator at 37 °C for 30 min. Then, the myeloid cell fraction was added onto EC layer and incubated for further 30 min at 37 °C. In a last step, all cells were stained with Hoechst 33342 (Sigma-Aldrich, Buchs, Switzerland), fixed in PFA (4%) and analyzed on a confocal microscope (Leica SP8 inverse CLSM). The quantification was performed using Fiji (ver. 1.53i, NIH, Bethesda, MD, USA) by counting the number of immune cells to endothelial cells (Immune cells/EC ratio).

### 2.4. Clonogenic Assay

To assess the potential of CD157^−^ and CD157^+^ HDMEC to form colonies in vitro, we performed a clonogenic assay. Sorted CD157^−^ and CD157^+^ HDMEC (600 cells each) at passage 0–1 were seeded into 6 wells coated with 0.01 % gelatin solution and cultivated in EGM-2MV medium (Lonza, Switzerland) at 37 °C for 14 days with medium change every other second day. At day 14, the medium was aspirated and the cells were washed once with PBS (Invitrogen, Zug, Switzerland). Next, 3 mL of 6% glutaraldehyde and 0.5% crystal violet solution (each diluted in water, all Sigma-Aldrich, Buchs, Switzerland) was added for 30 min to all wells. Cells were washed with tap water and dried at room temperature. Cell colonies were counted under a microscope (Nikon AG, Egg, Switzerland; Software: Nikon ACT-1 version 2.70). Images were processed with Photoshop 10.0 (Adobe Systems, Inc., Basel, Switzerland).

### 2.5. Treatment of EC with Cytokines In Vitro

For the treatment with cytokines, two distinct BEC populations (CD31^+^PDP^−^CD157^+^ and CD31^+^PDP^−^CD157^−^) as well as two distinct LEC populations (CD31^+^PDP^+^CD157^+^ and CD31^+^PDP^+^CD157^−^) were separated by sorting and used at passage 0–1. The cells were seeded into 6-well plates coated with 0.01% gelatin solution and cultured in EGM-2MV (Lonza, Basel, Switzerland) at 37 °C until reaching 80–90% confluency. Two distinct cytokines, recombinant human Tumor-Necrosis-Factor-alpha (TNF-α; Peprotech, Hamburg, Germany) applied at concentration 16 ng/mL, and recombinant human Interferon-gamma (IFN-γ; Peprotech, Hamburg, Germany) applied at concentration 5 ng/mL, were diluted in 2 mL of culture media, added and the cells, which were further cultivated in the incubator (37 °C, 5% CO_2_) for either 24 h, 48 h, or 72 h. At the mentioned time points, the treatment was stopped by removing the cytokine-media mixture and the treated cells were then used either for flow cytometric analysis or immunofluorescence staining.

### 2.6. Immunohistochemical Staining

Immunofluorescence staining on cryosections was performed as described in [20]. For immunofluorescence staining the following antibodies were used: anti-human CD31 (clone JC70A, 1:50, DAKO, Switzerland), anti-CD157 (clone 534509, 1:100, R&D, Abingdon, UK), anti-human PROX1 (polyclonal, 1:100, ReliaTech, Munich, Germany), anti-human LYVE1 (clone ab10278, 1:100, abcam, Cambridge, UK), anti-human Podoplanin (clone 18H5, 1:100, Santa Cruz, Heoidelberg, Germany), anti-human CD11b (clone M1/70, 1:50, BD Pharmingen, Allschwil, Switzerland), anti-human HLA-DR (clone: LN3, 1:50, BioLegend, Lucerne, Switzerland), anti-human CD54 (ICAM1) (clone: HCD54, 1:50, BioLegend, Lucerne, Switzerland), anti-rat CD11b/c (clone OX42, 1:50, BioLegend, Lucerne, Switzerland), anti-rat Myeloid Lineage Antibody-FITC (clone OX-82, 1:50, BioLegend, Lucerne, Switzerland), anti-rat Granulocytes (clone HIS48, 1:100, Heidelberg, Santa Cruz, Germany). As secondary antibodies, we used: anti-mouse Alexa Fluor 488, anti-rabbit Alexa Fluor 488, anti-mouse Alexa Fluor 568, anti-rabbit Alexa Fluor 568, anti-mouse Alexa Fluor 647 (all from Abcam). For double immunofluorescence, some of the primary antibodies were pre-labeled with Alexa 488, 647 or 555-conjugated polyclonal goat F(ab′)2 fragments, according to the manufacturer’s instructions (Zenon Mouse IgG Labeling Kit, Molecular Probes, Invitrogen, Zug, Switzerland). Matched isotype controls were used instead of primary antibodies at the concentration as specific antibodies: isotype control PE (clone MOPC-21, BD Pharmingen, Allschwil, Switzerland), isotype control AF488 (clone 11411, Novus, Manchester, UK), isotype control FITC (clone MOPC-173, BioLegend, Lucerne, Switzerland). Images were taken by a fluorescence microscope (Nikon AG, Egg, Switzerland; Software: Nikon ACT-1 version 2.70). Images were processed with Photoshop 10.0 (Adobe Systems, Inc., Basel, Switzerland).

### 2.7. Quantification of CD157 Expression on Blood and Lymphatic Capillaries

The following procedure was followed for evaluating stained 6–8 μm thick cryo-sections: normal juvenile/adult (j/a) and fetal (f) human skin biopsies were triple-stained for CD31^+^PDP^−^CD157^+^ (BEC) and CD31^+^PDP^+^CD157^+^ (LEC) and quantified using Fiji image analysis software (ver. 1.53i, NIH, USA). At least three sections from each biopsy were stained by triple immunofluorescence technique and five images were randomly obtained (n = 5 independent skin donors). The number of CD157-expressing capillaries was quantified as percentage of all blood (CD31^+^Podo^−^) and lymphatic (CD31^+^Podo^+^) capillaries in normal j/a and f human skin. The entire view field regions at 10× magnification images were counted (n = 10 j/a and n = 10 f skin biopsies). 

### 2.8. Preparation of vascDESS and Non-vascDESS

Collagen type I hydrogels were prepared as previously described [21]. In total, 5 × 10^4^ EC and 5 × 10^4^ fibroblasts (1:1 ratio) were resuspended in 1 mL collagen gel to generate prevascularized DESS (vascDESS). The non-vascularized controls (non-vascDESS) were prepared with 1 × 10^5^ fibroblasts without EC. All gels were placed in 6-well cell culture inserts with membranes of 3.0 μm pore size (BD Falcon, Kaiserlautern, Germany) and kept for 30 min at 37 °C in a humidified incubator containing 5% CO_2_. After a polymerization period, EGM-2MV (Lonza, Basel, Switzerland) was added to the upper and lower chambers of the well/insert and hydrogels were incubated for two weeks. Then, hydrogels were covered by keratinocytes (7.5 × 10^4^/gel), cultured for an additional week, and transplanted onto immuno-incompetent rats [21]. In total, three different skin cell donors (n = 3) were used for hydrogel preparation.

### 2.9. Transplantation of Tissue-Engineered Skin Substitutes

The surgical protocol was approved by the local Committee for Experimental Animal Research (Cantonal veterinary office Zurich, permission number ZH045/2019). Immuno-incompetent female nu/nu rats, eight- to ten-weeks-old (Envigo, Horst, The Netherlands) were anesthetized by inhalation of 5% Isoflurane (Baxter, Volketswil, Switzerland), and maintained by inhalation of 2.5% Isoflurane via mask. The dermo–epidermal skin substitutes were transplanted on full-thickness skin wounds created on the back of the rats. 

Following this, vascDESS (6 rats) and non-vascDESS (6 rats) were prepared using three independent donors for HDMEC, fibroblasts, and keratinocytes each (n = 3) and transplanted onto full-thickness skin defects prepared on the backs of the rats (12 rats in total) for one week. To prevent wound closure from surrounding rat skin, custom made steel rings (diameter 2.6 cm) were sutured into full-thickness skin defects using non-absorbable polyester sutures (Ethibond^®^, Ethicon, NJ, Somerville, USA). The transplants were then covered with a silicone foil (Silon-SES, BMS, USA), a polyurethane sponge (Ligasano, Ligamed, Innsbruck, Austria), a cohesive conforming bandage (Sincohaft, Theo Frey AG, Bern, Switzerland), and tape as wound dressing. Animals were euthanized using carbon dioxide and the transplanted skin analogs were harvested after 1 week by in toto excision and processed for immunohistochemical analysis.

### 2.10. Statistical Analysis

All results are reported as mean ±SD. Statistical analysis was performed with GraphPad Prism 4.0 (Graph Pad software, La Jolla, CA, USA). Comparison between two groups was performed using the two-tailed unpaired Student’s *t*-test and between multiple groups using two-way ANOVA with Bonferroni multiple comparisons test. * indicates *p*-value 0.01 to 0.05 (significant), ** indicates *p*-value 0.001 to 0.01 (very significant), *** indicates *p*-value 0.0001 to 0.001 (extremely significant), **** indicates *p*-value *p* < 0.0001 (extremely significant), ns indicates *p* ≥ 0.05 not significant.

## 3. Results

### 3.1. Both Blood and Lymphatic Capillaries of Juvenile/Adult and Fetal Skin Express CD157

**Figure 1:**
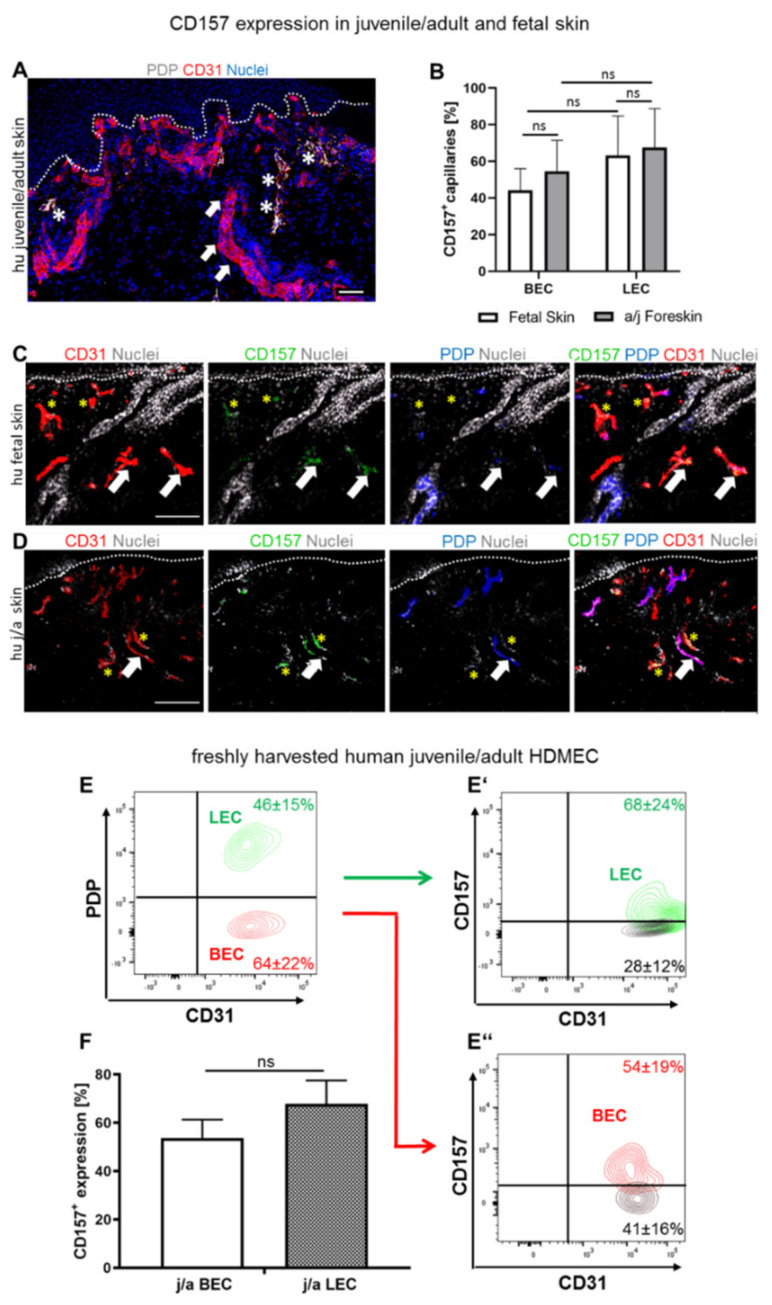
CD157 expression in human juvenile/adult (j/a) and fetal (f) skin. (**A**) Representative immunofluorescence image used for the differentiation between blood (CD31^+^/PDPN; white arrows) and lymphatic (CD31^+^/PDPN^+^; white asterisks) capillaries (scale bar: 50 µm). Dotted line indocate dermo-epidermal junction. (**B**) The fraction of CD157^+^ capillaries in fetal (f) and juvenile/adult (j/a) foreskin skin sections was assessed based on the total number of blood and lymphatic capillaries (n = 7). (**C**,**D**) Human f and j/a skin was stained for endothelial-specific CD31, CD157 and PDPN markers, (scale bars: 100 µm). Asterisks indicate CD31^+^PDP^-^CD157^+^ capillaries, white arrows point to CD31^+^PDP^+^CD157^+^ capillaries. Dotted lines delineate dermo-epidermal junction. (**E**–**F**) Sorting (**E**,**E″**) and quantification (**F**) of freshly isolated CD157^+^ and CD157^−^BEC and LEC using FACS sorting (*p* = 0.6144, ns; n = 7). Statistical tests were performed using two-way ANOVA.

Whereas blood capillaries were identified as CD31-positive and PDP-negative (CD31^+^/PDP^−^; yellow asterisk), lymphatic capillaries were stained positive for CD31 and PDP (CD31^+^/PDP^+^; white arrow) (Figure 1A). Then, the expression of CD157 was quantified on blood endothelial cells (BEC) and lymphatic endothelial cells (LEC) (Figure 1B), using triple immunofluorescence co-staining for CD31, podoplanin (PDP), and CD157, in human juvenile/adult (j/a), as well as fetal (f) skin (Figure 1C,D).

Overall, we detected a similar expressed level of CD157 on LEC (67.5 ± 21.2%) and BEC (54.4 ± 16.9%, *p* = 0.5985, ns) from human foreskin, as well as in fetal skin (LEC 63.1 ± 21.6%, BEC 44.2 ± 11.9%, *p* = 0.0660, ns) (Figure 1B). 

Figure 1C,D demonstrates the triple-immunofluorescence co-staining, used to assess the expression of CD157 profile on blood and lymphatic capillaries, in human juvenile/adult (Figure 1D) and fetal skin (Figure 1C). Accordingly, both skin types showed distinct types of blood (CD31^+^/PDP^−^/CD157^+^ and CD31^+^/PDP^−^/CD157^−^), as well as lymphatic capillaries (CD31^+^/PDP^+^/CD157^+^ and CD31^+^/PDP^+^/CD157^−^) (Figure 1C,D).

Further, to quantify CD157 expression in skin on a single-cell level, FACS analysis of freshly isolated HDMEC was performed (Figure 1E). The detailed gating strategy is described in the Supplementary Materials (Supplementary Figure S1). More specifically, the whole dermal fraction of j/a foreskin was freshly harvested, stained, and analyzed for CD31, PDP, and CD157 (Figure 1E). First, we assessed the ratio of LEC (46.1 ± 15.2%) to BEC (64.3 ± 22.2%) in the freshly isolated human juvenile/adult dermal fraction (Figure 1E). As further confirmed, PDP and PROX1 stained the same LEC and, therefore, PDP and PROX1 were used as interchangeable LEC markers in this study (Figure S2). Further, we detected similar CD157 expression on j/s LEC (67.9 ± 23.6%) and j/a BEC (53.7 ± 18.6%; *p* = 0.6144, ns) (Figure 1F).

Due of the small size of human fetal skin biopsies, we were unable to isolate sufficient numbers of fetal HDMEC from the dermis for FACS analysis. Therefore, isolated fetal HDMEC were first expanded in vitro and analyzed by flow cytometry at passage 1 (P1). The analysis of cultured fetal HDMEC (P1) demonstrated that 10.1 ± 2.9% of fetal BEC (fBEC) expressed CD157, whereas 41.1 ± 15.1% fetal LEC (fLEC) expressed this marker (Supplementary Figure S3). Thus, the CD157 expression was significantly enhanced in cultured fLEC populations compared to fBEC (*p* < 0.0001) (Supplementary Figure S3).

Similarly, the analysis of CD157 expression profile of cultured j/a HDMEC revealed that only 21.06 ± 11.6% of cultured (P1) dermal j/aBEC expressed this marker, whereas 58.03 ± 16.8% (*p* < 0.000) of j/aLEC were CD157 positive (Supplementary Figure S4). As control, HUVEC were used. Only a few HUVEC expressed CD157 (3.13 ± 1.71%) (Supplementary Figure S4).

### 3.2. CD31^Low^ and CD31^High^ j/a HDMEC Differ in Their Levels of CD157 Expression

**Figure 2:**
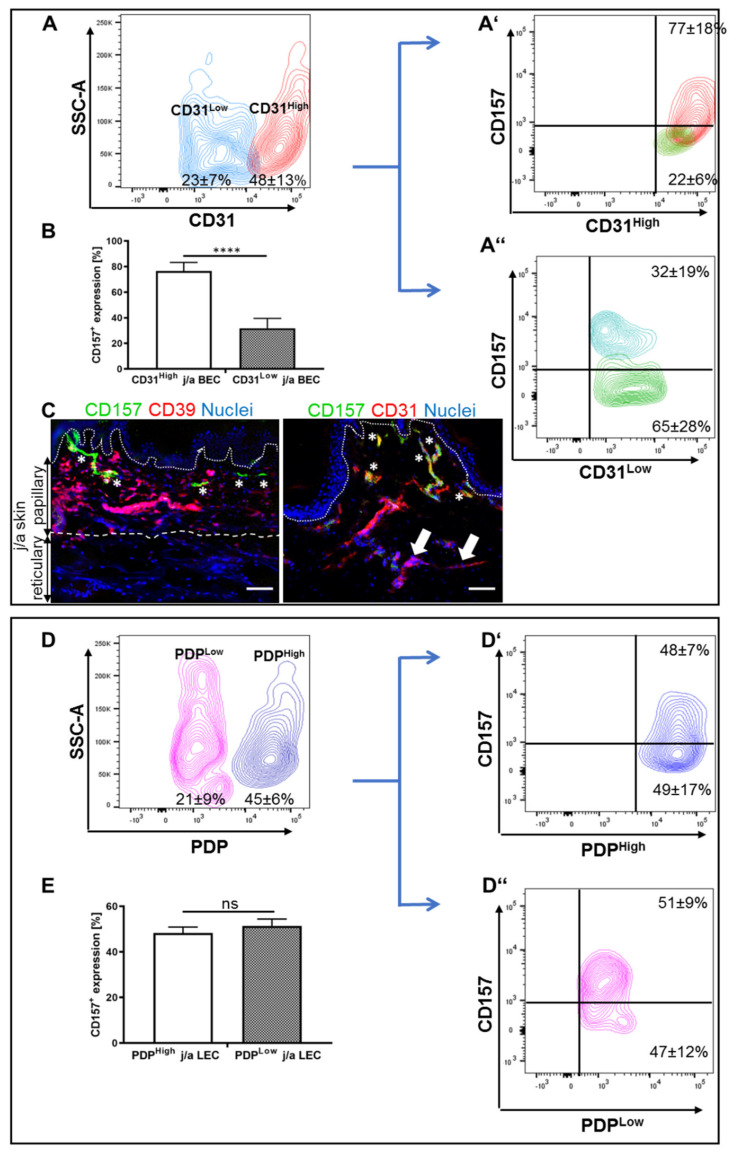
CD157 expression on CD31^High/Low^ and podoplanin^High/Low^ expressing freshly isolated HDMEC (**A**,**B**) The whole HDMEC population was separated into two distinct fractions: CD31^High^ and CD31^Low^ expressing subpopulations. (**A′**,**A″**,**B**) Representative FACS dot plots (**A**,**A″**) and quantification (**B**) of freshly isolated and separated cells. Whereas CD31^High^ HDMEC showed enhanced expression of CD157^+^, CD31^Low^ subpopulation showed a reduced expression of CD157 (**** *p* = 0.0010 extremely significant; n = 7). (**C**) Immunofluorescence co-staining for CD39, a papillary fibroblast marker (red) and CD157 (green) in j/a skin showing the presence of CD157 expressing capillaries (asterisks) in CD39-positive papillary dermis (**C**). Further, co-staining for CD31 (red) and CD157 (green) demonstrates CD157^+^CD31^High^ capillaries (asterisks) in the upper, papillary dermis, whereas CD157^-^CD31^Low^ capillaries (arrows) are predominantly located in lower, reticular dermal parts. (**D**,**D″**) HDMECs show distinct separation into PDP^High^ and PDP^Low^ expressing subpopulations. (**E**) Quantification of CD157 expression on PDP^High^ and PDP^Low^ HDMEC revealed no statistically significant differences between those two subpopulations (*p* = 0.530, ns; n = 7). Dotted lines delineate the dermo–epidermal junction. Dashed lines separate the papillary and reticular dermis. Statistical tests were performed using two-way ANOVA.

Next, we sought to perform in-depth analysis of CD157 expression profiles with regard to CD31 levels in freshly isolated BEC and LEC fractions of human j/a skin (Figure 2A). Accordingly, we detected two separate CD31-positive and podoplanin-negative (CD31^+^PDP^−^) BEC populations: CD31^Low^, which accounted for 23.4 ± 7.3% of all HDMEC cells and CD31^High^ cells, which represented 48.4 ± 13.5% of all HDMEC cells (Figure 2A). 

Where the majority of CD31^High^ BEC (77 ± 18%) expressed CD157, this number significantly decreased to 32 ± 19% for CD31^Low^ BEC (Figure 2A,A″,B, *p* = 0.0010). Moreover, we detected CD157^+^ capillaries primarily in CD39-positive upper papillary dermis, whereas they were almost absent in the lower reticular dermis (Figure 2C; Supplementary Figure S5A–C). Although we also detected co-stained CD31^+^CD157^+^-positive capillaries in the papillary dermis, only CD31^+^CD157^−^ capillaries were identified in lower reticular parts of the dermis (Figure 2C).

Further, we investigated, in more detail, the expression profile of CD157 on freshly isolated CD31^+^PDP^+^ LEC in j/a skin (Figure 2D). The analysis revealed that the population of LEC expressing high levels of PDP (CD31^+^/PDP^High^), previously described as capillary-derived LEC [22], accounted for 45 ± 6% of all LEC cells, whereas CD31^+^/PDP^Low^ LEC cells, derived from precollector lymphatic vessels [22], represented only 21 ± 9% (Figure 2D,D″) of analyzed cells. Regarding the expression level of CD157 on PDP^Low^ and PDP^High^ LEC, we detected a similar level in both subpopulations: 48 ± 7% and 51 ± 9%, respectively (Figure 2E, *p* = 0.5308, ns). Further, both CD31^+^/PDP^Low^ and CD31^+^/PDP^High^ showed similar levels of CD31 (data not shown).

### 3.3. CD157 Expression Is Reduced after Prolonged Culturing In Vitro

**Figure 3:**
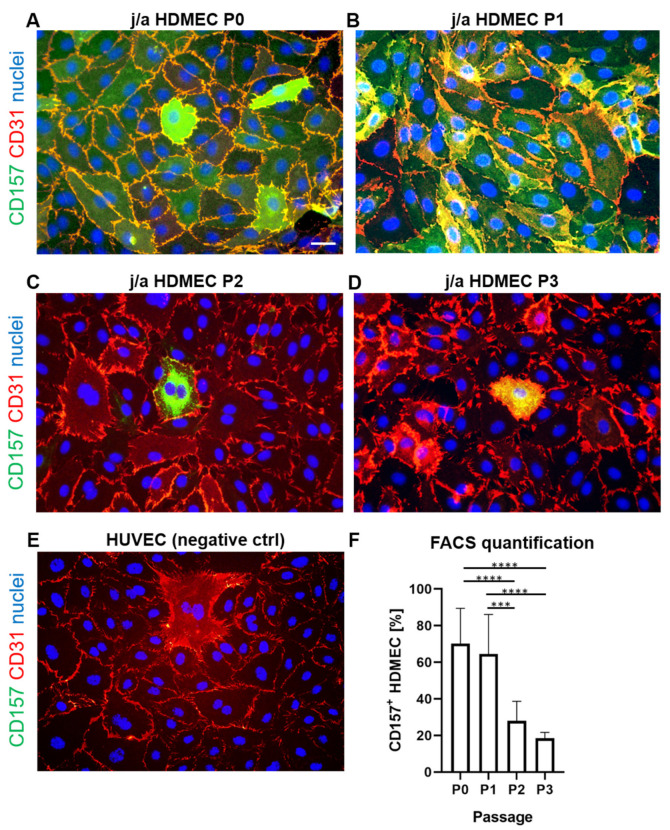
Expression profile of CD157 on cultured endothelial cells in vitro (**A**–**E**) HDMEC cultured at passages 0 (**A**), 1 (**B**), 2 (**C**), and 3 (**D**) were stained for CD31 (red), CD157 (green), and counterstained for Hoechst (blue). HUVEC (**E**) were used as negative control. (**F**) A quantification using cell expression measured by flow cytometry, showed a similar high expression of CD157 with 69.2 ± 23.7% expressing cells at P0 and 64.5 ± 21.6% at P1; *p* = 0.96, ns). Further, strong decrease in CD157 expression levels was observed at P2 in vitro (27.9 ± 10.8% vs. P0; **** *p* < 0.0001 extremely significant), and at P3 (18.5 ± 3.2% vs. P0; **** *p* < 0.0001 extremely significant) (n = 10). *** *p* < 0.001 extremely significant. Statistical tests were performed using two-way ANOVA.

Next, we examined the CD157 expression profile, after short and long cultivation of juvenile/adult (j/a) HDMECs in vitro (Figure 3). Immunofluorescence analysis of in vitro cultured HDMEC revealed stable and relatively high CD157 expression at low j/a HDMEC passages (P0–P1) in vitro, whereas prolonged cultivation (P2–P3) resulted in a low expression level of this marker in vitro (Figure 3A–D). HUVEC was used as a negative control (Figure 3E).

To quantify CD157 expression on a single-cell level, we also performed FACS analysis (Figure 3F). Accordingly, j/a HDMEC at P0 and P1 showed similarly high levels of CD157 expression with 69.2 ± 23.7% (P0) and 64.5 ± 21.6% (P1); *p* = 0.96, ns). However, we detected a sharp decrease in CD157 expression on j/a HDMEC, starting at P2 in vitro (27.9 ± 10.8%; *p* < 0.0001; vs. P0), and even further reduction at P3 (18.5 ± 3.2%; *p* < 0.0001; vs. P0).

We next separated cultured j/a HDMEC into BEC and LEC and assessed CD157 expression on those fractions (Supplementary Figure S4A). The FACS analysis revealed that only 21.06 ± 11.6% of all dermal j/aBEC expressed the CD157 marker, whereas, in contrast, 58.03 ± 16.8% (*p* < 0.000) of j/aLEC were CD157 positive for this marker. Further, only a few HUVEC expressed CD157 (3.13 ± 1.71%) (<0.0001 vs. j/aLEC and *p* = 0.0226 vs. j/aBEC) (n = 20).

### 3.4. Proliferation and Clonal Expansion of CD157^+^ and CD157^−^ HDMEC In Vitro

**Figure 4:**
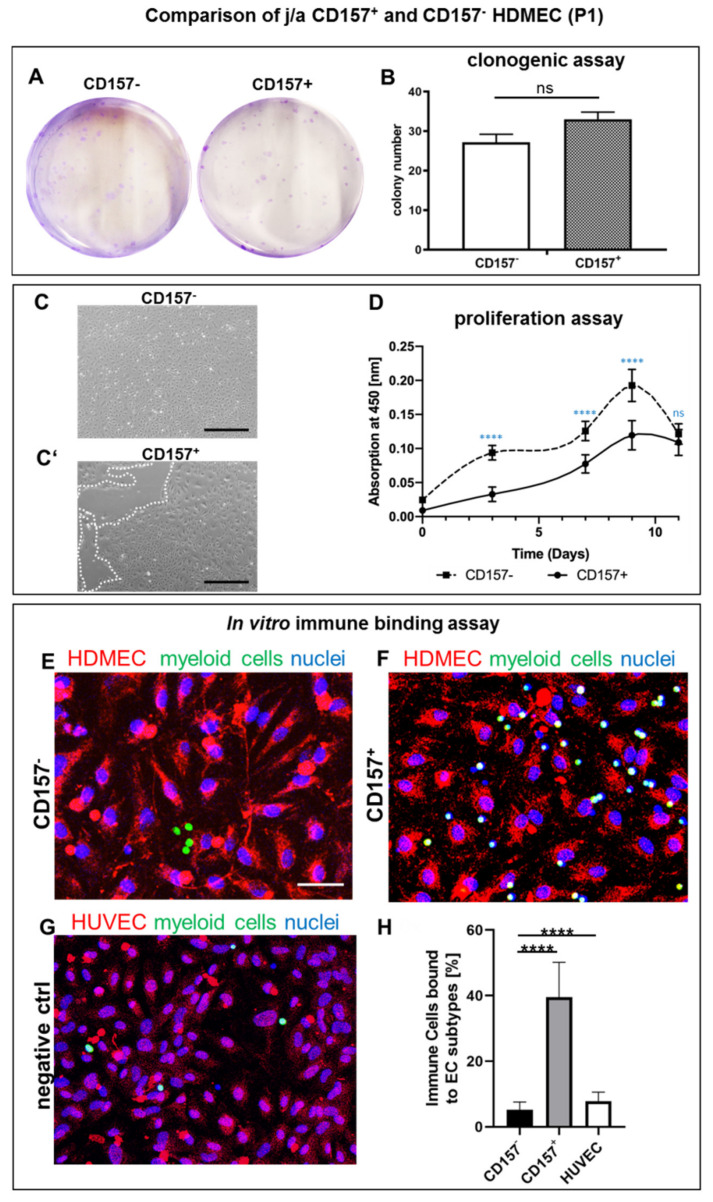
Comparison of clonogenic, proliferation, and immune binding potential of j/a CD157^+^ and CD157^−^ HDMEC in vitro (**A**,**B**) CD157^+^ and CD157^−^ HDMEC gave rise to similar colony numbers after sorting (*p* = 0.0589, ns; n = 5) (**C**,**C**′). Whereas CD157^−^ HDMEC rapidly reached a confluent monolayer (C), CD157^+^ HDMECs demonstrated only a partial confluency (**C**′). White dashed lines delineate the areas not covered by cells (n = 5, scale bar: 50 µm). (**D**) Colorimetric proliferation assays with CD157^+^ and CD157^−^ using 1 × 10^4^ seeded cells in 24-well plates were conducted at d = 0, 3, 7, 9, 11 (n = 5, *p* ≤ 0.0001). (**E**–**H**) In vitro immune binding assay reveals strong adhesion of CD11b expressing myeloid cells to CD157^+^ (39.5 ± 10.7%), but only weak adhesion to CD157^−^ (5.2 ± 2.4%, **** *p* < 0.0001 extremely significant) or HUVEC (7.8 ± 2.8%, **** *p* < 0.0001 extremely significant). Statistical tests were performed using two-way ANOVA.

Since CD157 was described as a marker of tissue-resident EC stem cells in mice with enhanced proliferation and clonogenic potential [5], we compared the respective characteristics between CD157^−^ and CD157^+^ HDMEC derived from j/a foreskin. Representative images of colony-forming assays are shown in Figure 4A. We detected similar numbers of colonies derived from CD157^−^ (27.2 ± 5.0) and CD157^+^ (33.0 ± 4.5; *p* = 0.059, ns) EC (Figure 4A,B). 

Further, we investigated the relative proliferation rate of CD157^−^ and CD157^+^ HDMEC fraction, using a colorimetric proliferation assay. Phase contrast images, in Figure 4C,C′, demonstrate that using the same seeding density of HDMEC from independent skin donors, CD157^−^ cells were rapidly confluent already at P1, whereas CD157^+^ HDMEC were not. Further, applying a standard proliferation assay, we detected that CD157^+^ HDMEC showed constantly lower cycling rates, as compared to the CD157^−^ population during the time course in vitro. Eventually, at the final time point, at day 11, we observed a sharp decrease in cycling rates for both populations, suggesting saturated proliferation (Figure 4D).

Further, we confirmed that monolayer cultivated j/aHDMECs were viable and proliferated in vitro in confluent 2D, as shown by Ki67 and CD31 co-stainings (Supplementary Figure S4B,C). Further, monolayer EC displayed typical “cobblestone” endothelial morphologies and showed well-developed focal contacts.

### 3.5. Myeloid Cells Expressing CD11b Adhere Specifically to CD157^+^ HDMEC In Vitro

As CD157 is involved in chemotaxis and the rolling/diapedesis of immune cells, we assessed the interactions between CD157 present on HDMEC and CD11b integrin expressed on myeloid cells, using a specific immune-binding assay in vitro (Figure 4E–H; Supplementary Figure S6). CD157^−^ EC fraction and/or HUVEC were used as a negative control.

The ratio of CD11b myeloid cells, that specifically adhered to the cell surface of distinct HDMEC, was quantified (Supplementary Figure S6) and is shown in Figure 4E–H. Our results indicate that only a few CD11b^+^ immune cells (green) bound to CD157^−^ HDMEC (Figure 4E–H; 5.2 ± 2.4%), as compared to CD157^+^ HDMEC fraction (Figure 4F,H; 39.5 ± 10.7%, *p* < 0.0001), which exhibited the highest fraction of adhered myeloid cells. Moreover, HUVEC also demonstrated a low binding of myeloid cells to their surface (Figure 4G,H; 7.8 ± 2.8%).

### 3.6. Pro-Inflammatory Cytokines Stimulate Differently HLA-DR and ICAM1 Expression in CD157^−^ and CD157^+^ BEC/LEC Populations In Vitro

Since CD157 plays a role in immune cell trafficking [12], we also sought to assess the expression profile of human leukocyte antigens (HLA-DR) and other adhesion molecules, including ICAM1 in sorted CD157^+^ and CD157^−^ BEC and LEC, before and after cytokine-stimulation in vitro. Both HLA-DR [23] and adhesion molecules [24] are both important for the activation and homing of immune cells and promoting inflammation.

**Figure 5:**
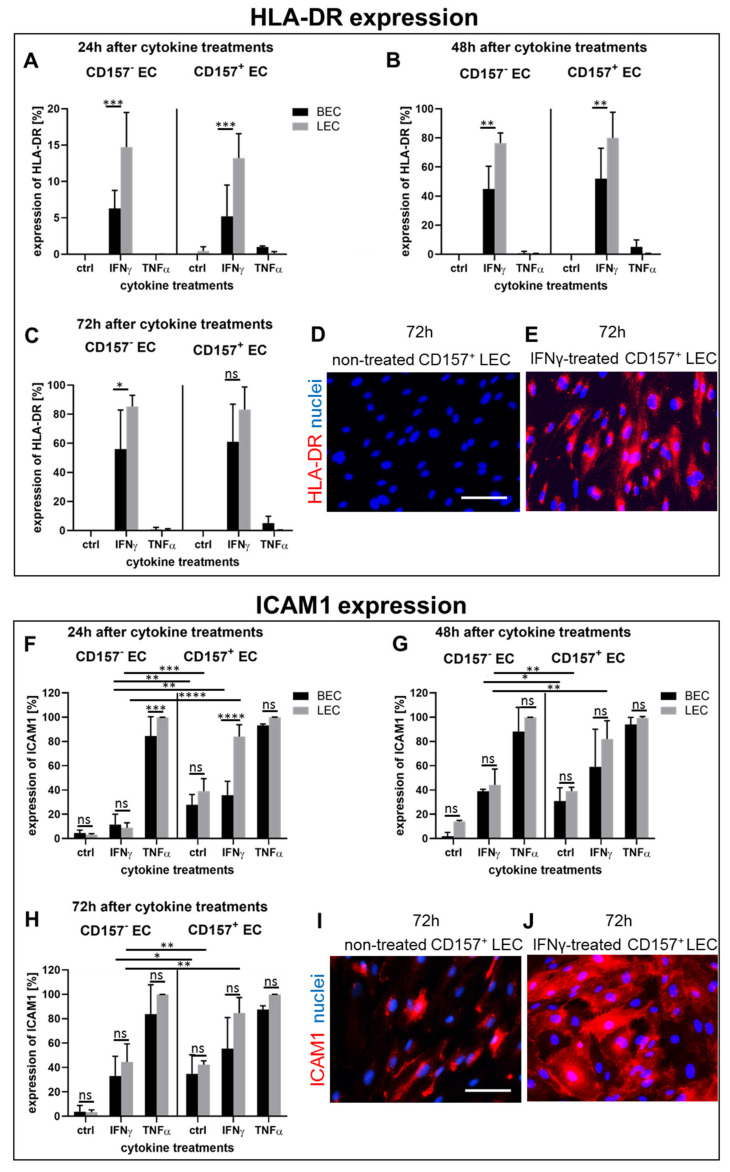
Induction of HLA-DR and ICAM1 expression in CD157^−^ and CD157^+^ BEC and LEC in vitro (**A**–**E**) The protein expression of HLA-DR before and after 24-h (**A**), 48-h (**B**), and 72-h (**C**,**E**) stimulation of CD157^−^ and CD157^+^ BEC and LEC with IFNγ and TNFα in vitro (n = 3). The expression level was assessed by flow cytomeric analysis (**A**–**C**) and immunofluorescence staining (**D**,**E**). (**F**–**J**) The expression of ICAM1 before after 24-h (**F**), 48-h (**G**), and 72-h (**H**,**J**) stimulation of CD157^−^ and CD157^+^ BEC and LEC with IFNγ and TNFα in vitro. The expression level was assessed by flow cytometric analysis (**A**–**C**) and immunofluorescence staining (**I**,**J**) (n = 3). **** indicates *p* < 0.0001 extremely significant, *** indicates *p* < 0.001 extremely significant, ** indicates *p* < 0.01 very significant, * indicates *p* < 0.05 significant, ns indicates *p* ≥ 0.05 not significant. Statistical tests were performed using two-way ANOVA. Scale bars: 50 µm.

The basal expression profile of HLA-DR in CD157^+^ or CD157^−^ BEC/LEC, before treatment, was not detectable, using either flow cytometric analysis (Figure 5A–C) or immunofluorescence staining (Figure 5D). However, 24-h long stimulation with IFNγ induced a significantly higher HLA-DR expression in CD157^−^ LEC (14.7 ± 4.8) than in CD157^−^ BEC (6.3 ± 2.5; *p* = 0.0006), respectively. Similarly, CD157^+^LEC (13.2 ± 2.2) demonstrated higher IFNγ-induced HLA-DR expression than CD157^+^ BEC (5.21 ± 3.3; *p* = 0.001). In contrast, TNFα treatment did not show any effect on CD157^−^ BEC and LEC (Figure 5A), whereas it demonstrated a weak induction in CD157^+^ LEC (0.15 ± 0.2) versus CD157^−^ BEC (1.0 ± 0.1; *p* = 0.9645, ns). Accordingly, the IFNγ stimulation showed similar effects in both CD157^−^ and CD157^+^ EC fractions.

Both 48-h and 72-h treatment modalities demonstrated a potent induction of HLA-DR; however, this was without significant differences between CD157^−^ and CD157^+^ EC fractions. In particular, 48-h long IFNγ-stimulation up-regulated HLA-DR expression in CD157^−^ BEC (47.9 ± 20.6), and even more, increased up-regulation in CD157^−^ LEC (76.5 ± 6.9; *p* = 0.0021). Similarly, HLA-DR induction after IFNγ-stimulation (48 h) was observed in CD157^+^ BEC (49.1 ± 25.8) and CD157^+^ LEC (80.0 ± 17.6; *p* = 0.0067). In comparison, 72 h IFNγ-stimulation induced the highest HLA-DR expression in CD157^−^ BEC (56.0 ± 26.8) and CD157^−^ LEC (85.3 ± 7.7; *p* = 0.0021), as well as in CD157^+^ BEC (61.1 ± 25.8) and CD157^+^ LEC (83.0 ± 16.6; *p* = 0.135, ns). Similarly, we observed a negative HLA-DR expression using immunofluorescence staining for untreated cells (Figure 5D) and a positive staining in 72 h IFNγ-stimulated CD157^+^ LEC (Figure 5E).

In contrast, TNFα cytokine stimulated HLA-DR expression only in CD157^+^ EC, showing an almost identical stimulation pattern after 24 h, 48 h and 72 h, but to a lesser extent than IFNγ treatment. Moreover, we detected significant differences in the basal protein expression level of ICAM1, between CD157^−^ and CD157^+^ HDMEC fractions (Figure 5F–H). Whereas unstimulated (ctrl) CD157^−^ were only weakly positive for ICAM1 (CD157^−^ BEC (2.6 ± 3.7) CD157^−^ LEC (6.8 ± 0.6)), the basal expression profile of ICAM1 in CD157^+^ cells showed enhanced expression values (CD157^−^ BEC (23.4 ± 1.4), and CD157^−^ LEC (40.2 ± 3.6)).

Both IFNγ and TNFα cytokines significantly induced ICAM1 protein expression, in particular, within the CD157^+^ HDMEC fraction, with expression peak levels reached after 72 h (Figure 5H).

Accordingly, IFNγ increased the expression of ICAM1 after 24 h in CD157^−^ BEC (11.6 ± 8.5) and CD157^−^ LEC (9.0 ± 4.0; *p* = 0.974, ns), as well as in CD157^+^ BEC (35.8 ± 11.4) and CD157^+^ LEC (84.0 ± 9.7; *p* < 0.0001). Further, 48-h long IFNγ treatment resulted in even higher ICAM1 expression in CD157^−^ BEC (38.9 ± 1.6) and CD157^−^ LEC (44.2 ± 13; *p* = 0.9563, ns), and enhanced values in CD157^+^ BEC (59.8 ± 34.9) and CD157^+^ LEC (82.0 ± 15.1; *p* = 0.148, ns). 

Notably, 72 h of IFNγ stimulation activated similarly high ICAM1 expression values in CD157^−^ BEC (38.9 ± 1.6) and CD157^−^ LEC (44.2 ± 13; *p* = 0.9563, ns), as well as in CD157^+^ BEC (59.8 ± 34.9) and CD157^+^ LEC (82.0 ± 15.1; *p* = 0.148, ns).

Moreover, TNFα cytokine simulation induced the highest expression of ICAM1 and confirmed increased capability of ICAM1 protein induction in CD157^+^ HDMEC population. Accordingly, 24-h long TNFα treatment increased the expression of ICAM1 in CD157^−^ BEC (84.6 ± 21.8) and CD157^−^ LEC (99.9 ± 2.0; *p* = 0.1109, ns), as well as in CD157^+^ BEC (93.3 ± 1.8) and CD157^+^ LEC (99.9 ± 1.1; *p* = 0.728, ns). After 48 h TNFα stimulation, ICAM1 expression values were as follows: CD157^−^ BEC (85.2 ± 19.9) and CD157^−^ LEC (99.2 ± 1.3; *p* = 0.6733, ns), as well as in CD157^+^ BEC (94.1 ± 5.8) and CD157^+^ LEC (99.3 ± 1.1; *p* = 0.9547, ns). Moreover, 72-h long TNFα simulation resulted in the highest ICAM1 protein expression in CD157^−^ BEC (87.3 ± 2.9) and CD157^−^ LEC (99.8 ± 1.2; *p* = 0.4737 ns). Similarly, high values were found in CD157^+^ BEC (83.8 ± 24.1) and CD157^+^ LEC (99.8 ± 1.5; *p* = 0.6871, ns).

Further, we also detected enhanced expression of CD62P (P-selectin) on CD157^+^ BEC, as compared to CD157^−^ BEC, after TNFα stimulation after 24 h, 48 h, and 72 h (data not shown). In contrast, we observed continuously high levels of ICAM2 (CD102) in all analyzed EC fractions, before and after cytokine treatments (data not shown).

### 3.7. CD157 Interacts with Myeloid Cells in Human Skin and Skin Substitutes In Vivo

**Figure 6:**
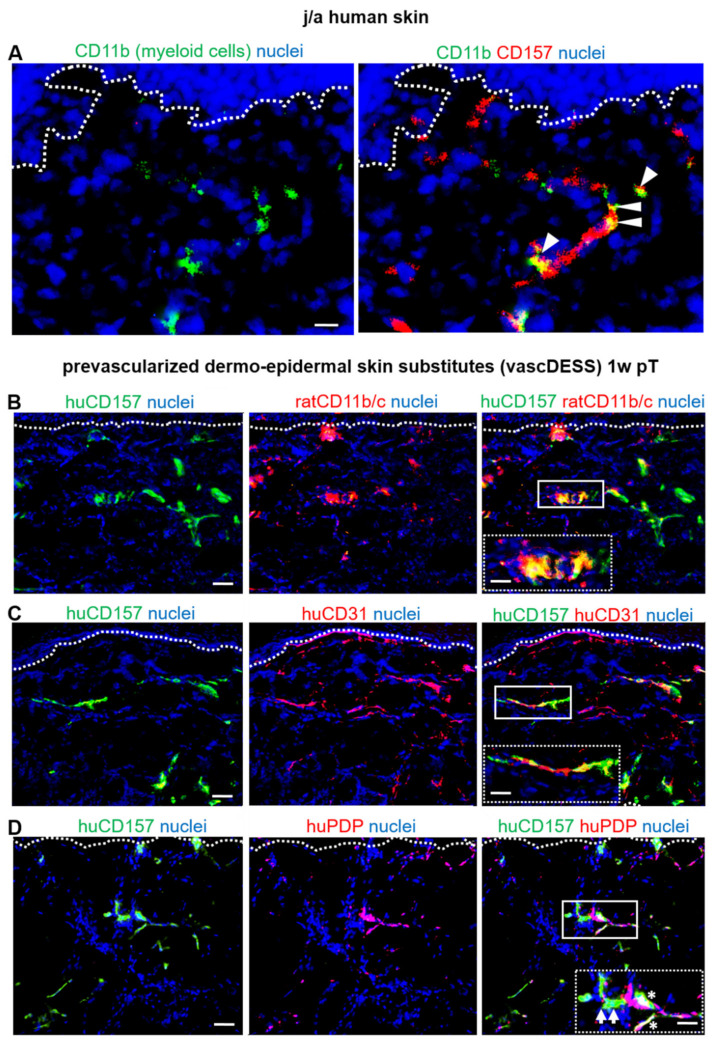
CD157 expression pattern on prevascularized dermo-epidermal skin substitutes (vascDESS) in vivo (**A**) Human myeloid CD11b-expressing cells in j/a foreskin sections co-localized with CD157^+^ capillaries (white arrowheads). (**B**) Multiple rat CD11b/c myeloid cells infiltrated vascDESS with the vast majority of them binding to CD157^+^ capillaries in dermal part in vivo after 1 week post-transplantation (pT). Insets represent magnified images of respective framed areas. (**C**,**D**) Immunofluorescence staining of in vivo transplants 1 week (w) post-transplantation (pT) confirm huCD157 expression on all human CD31 capillaries (n = 3 independent donors (**C**), including podoplanin-negative (PDP^−^, white arrows) blood and podoplanin-positive (PDP^+^, white asterisks) lymphatic capillaries (n = 5). Scale bars: 50 µm (images) and 100 µm (inserts). Dotted lines delineate dermo-epidermal junction.

Further, we examined the interactions of myeloid cells and CD157^−^/CD157^+^ HDMEC lining blood and lymphatic capillaries in native human skin and in bioengineered pre-vascularized dermo–epidermal skin substitutes (vascDESS) (Figure 6). We observed that CD11b^+^ myeloid cells were found exclusively associated with CD157^+^ capillaries (white arrowhead) in normal human skin (Figure 6A).

We then sought to determine if these cell–cell interactions can also occur in vascDESS, in a rat model. Indeed, one week post-transplantation, we detected numerous CD11b/c rat myeloid cells that infiltrated the human skin transplant, with the vast majority of them detected in close proximity to human CD157^+^ capillaries (Figure 6B). The inset in Figure 6B shows the specific adherence of those two cell types at higher magnification. 

Further, we demonstrated CD157 expression on all human CD31^+^ capillaries in vascDESS in vivo (Figure 6C). Moreover, we confirmed CD157 expression on human podoplanin-positive lymphatic (PDP^+^; asterisks) and blood capillaries (PDP; arrows) in vascDESS in vivo (Figure 6D).

## 4. Discussion

We identified CD157 as a marker with specific and crucial roles in the regulation of skin innate immunity. In particular, our work demonstrates that CD157 (1) is constitutively expressed at vascular blood (BEC), and lymphatic endothelial cells (LEC), in vitro and in vivo, (2) affect the levels of immune adhesion molecules, following stimulation with pro-inflammatory cytokines, (3) orchestrates adhesion of myeloid cells in vitro and in vivo. Specific aspects of this study require detailed consideration. 

Insufficient vascularization represents the major obstacle in the tissue engineering of large skin constructs. Rapid proliferation rate, stable endothelial phenotype, high vasculogenic potential, and appropriate interactions of EC with other cell types, during skin wound healing, in particular, immune cells, are requirements for a successful vascularization and skin regeneration process in vivo. Since CD157 has been reported to play a crucial role in the mouse neo-vascularization [25], we sought to investigate its role in human skin. 

We detected, here, a relatively high expression of CD157 in human fetal and adult blood (BEC) and lymphatic endothelial cells (LEC) in the dermis. In this line, the study of Iba et al., also reported high expression of CD157 in a mouse skin endothelial cell side population (SP-EC), representing a vascular stem cell reservoir [25]. However, the data of both studies are not directly comparable, as we did not apply, in this study, the Hoechst efflux assay to specify the SP-EC of human skin [25]. 

Further, Wakabayashi et al. identified CD157 expressing EC, exclusively, in large-diameter vessels, in different mouse organs, but not in small-diameter capillaries [5]. Our work, however, confirmed the high expression level of CD157 in HDMEC lining small-diameter capillaries of human skin in situ. This data comparison indicates that there is a strong variation in the expression of CD157, concerning different vessel types, in various organs of mice and humans. 

With regard to clonal expansion potential, we did not detect any differences between CD157-positive and -negative HDMEC fractions. These results are different to findings reported by Wakabayashi et al. for mouse organs [5]. However, further stem cell assays are required to investigate the stem cell potential of human CD157^+^ EC in more detail.

We observed, in this study, that the CD157^+^ HDMEC was the slower proliferating cell fraction, as compared to CD157^−^ cells. These results differ from the findings described in a mouse study, showing enhanced proliferative potential of CD157^+^ mouse EC, harvested from different organs [5]. Importantly, we have observed, in this study, that sorted CD157^+^ showed lower attachment to coated cell culture plates. We speculate that the antibody used for staining and sorting of distinct fractions possibly masked CD157 epitopes, leading to lower attachment of CD157^+^ HDMEC to coated plates afterwards, and, consequently, lower values in proliferation assay. Indeed, previous reports suggested an important functional link between high expression of CD157 and the increased cell attachment to ECM, spreading, and motility of cancer cells, pointing to an important function of CD157 in cell adhesion and migration [3]. 

Moreover, it was shown that the knockdown of CD157 in human ovarian cancer cells changed their morphology and cytoskeleton organization, and attenuated the activation of intracellular signaling that impaired their binding to different matrix proteins, such as fibronectin, gelatin, collagen type I, laminin I, and others [3]. Based on these data, we assumed that the masked CD157 epitopes, on freshly sorted HDMEC, partially blocked the CD157 receptor function, and, consequently, attenuated cell adhesion, spreading, and proliferation of the CD157^+^ cell fraction. 

Concerning the CD157 expression during in vitro cell expansion, we detected, in this study, a stable expression of CD157 at early passages (0–1), whereas extended cell culturing and cell passaging led to reduced expression of this marker. These findings are crucial when designing in vitro assays using CD157-positive cells, since already, at passage two, HDMECs exhibited a significantly reduced expression. Other groups also reported a down-regulation of other glycoprotein receptors and stem cell receptors during in vitro expansion [26,27]. However, so far, no specific studies have been performed on human CD157 expression profile in cultured HDMEC.

Our study demonstrates that enhanced expression of CD157 is accompanied by elevated levels of CD31. Importantly, CD31 is also known as platelet endothelial cell adhesion molecule 1, and thus, contributes to transendothelial leukocyte migration, cell–cell adhesion, and antiapoptotic signaling of EC [28]. In particular, we have shown that CD157 is highly enriched in the population of skin EC, which express high levels of CD31 (CD31^High^), whereas only low levels of CD157 are detected in CD31^Low^ HDMEC population. 

Further, we detected CD31^High^CD157^+^ capillaries, predominantly, in the CD39-positive upper (papillary) dermis, located close to the epidermis. These findings are indicative of potential involvement of CD157 in myeloid cell recruitment to the upper dermis of human skin. Consequently, these findings imply a functional heterogeneity of dermal capillaries, linked to their distinct immune functions in the papillary versus reticular dermis. 

Importantly, the process of inflammation leads to activation of vascular HDMEC that contributes to vascular leakage and the recruitment of leukocytes. These processes have been described as being particularly active in upper parts of the dermis, in the course of multiple inflammatory skin disorders, including psoriaris [29], eczema [30], atopic dermatitis [31], dermatoses [32], rosacea [33], and acute generalized exanthematous pustulosis (AGEP) induced by drugs [34,35]. In addition, neutrophil migration, and its accumulation in papillary dermis in active psoriatic lesions, has an established role in the pathophysiology of psoriasis [29,36].

TNF-α is one of the chemokines generated during the inflammatory phase of wound healing that provokes a robust up-regulation of the key leukocyte adhesion receptors, such as ICAM1, ICAM2, CD54 and CD62P [37] and, thus, plays a pivotal role in the pathogenesis of an early shock state (i.e., hypotension, fever), inducing increased inflammatory cell extravasation tissue damage by acting on endothelial cells [38]. Another inflammatory cytokine, IFNγ, further enhances endothelial activation induced by TNF-α, and regulates major histocompatibility complex (MHC) expression [39]. Therefore, both cytokines are implicated in allograft and xenograft rejection [40], and were applied in this study.

Interestingly, we detected enhanced protein expression of those adhesion molecules, in either unstimulated or cytokine-stimulated CD157-positive HDMEC, in particular, LEC. These findings are in accordance with those reported regarding the ability of human LEC to express increased levels of cytokine-induced cell adhesion molecules in culture [37]. However, this particular study did not investigate the co-expression of CD157 marker and, consequently, did not compare the levels of cell adhesion molecules between CD157^+^ and CD157^−^ fraction [37].

In particular, LEC residing in the papillary dermis were described to act as main binding sites for the myeloid cells. In a previous study, Yamamoto et al. reported that ICAM-1 and endothelial leukocyte adhesion molecule-1 (E-selectin) were predominantly expressed on HDMEC of microvessels in the papillary dermis of human psoriatic skin lesions [41]. Further, Groeger et al. analyzed inflamed skin samples and cytokine-stimulated organ-cultured skin and also detected a subset of “activated” HDMEC, expressing high levels of adhesion molecules within the papillary dermis [42]. However, none of the published reports studied a possible role of CD157 in those processes.

Furthermore, we observed that CD157 was also present on the bioengineered capillaries, when incorporated into prevascularized dermo–epidermal skin substitutes (vascDESS) and transplanted on rats. Importantly, we detected a high binding capacity of myeloid cells to those CD157-positive blood and lymphatic capillaries, located predominantly in upper parts of the dermis of vascDESS. These data imply that there might be a direct link between enhanced expression of CD157 and high levels of cell adhesion molecules, expressed by those HDMEC that result in increased binding of myeloid cells, including monocytes/macrophages and neutrophils in vivo. Collectively, CD157 represent a potential future target for anti-inflammatory therapies.

## Data Availability

The data that support the findings of this study are available on request from the corresponding author.

## Supplementary Materials

The following supporting information can be downloaded at: https://www.mdpi.com/article/10.3390/biomedicines10030676/s1, Figure S1: Gating strategy for flow cytometric analysis of HDMEC; Figure S2: Purity of sorted j/a BEC and LEC; Figure S3: CD157 expression in cultured fetal HDMEC (P1); Figure S4: CD157 expression in cultured juvenile/adult HDMEC (P1); Figure S5: CD157 expression in papillary/reticular dermis of human skin; Figure S6: Quantification of immune binding of CD11b immune cells to CD157^+^ endothelial cells.
