# Supplementary material for: Expression Profile of CD157 Reveals Functional Heterogeneity of Capillaries in Human Dermal Skin
Source: Biomedicines. 2022 Mar 15;10(3):676. doi: 10.3390/biomedicines10030676 (PMC8945771; doi:10.3390/biomedicines10030676)
Supplement: Supplementary file 1 [file biomedicines-10-00676-s001.zip › biomedicines-1585163-supplementary.pdf]

Supplementary Figures

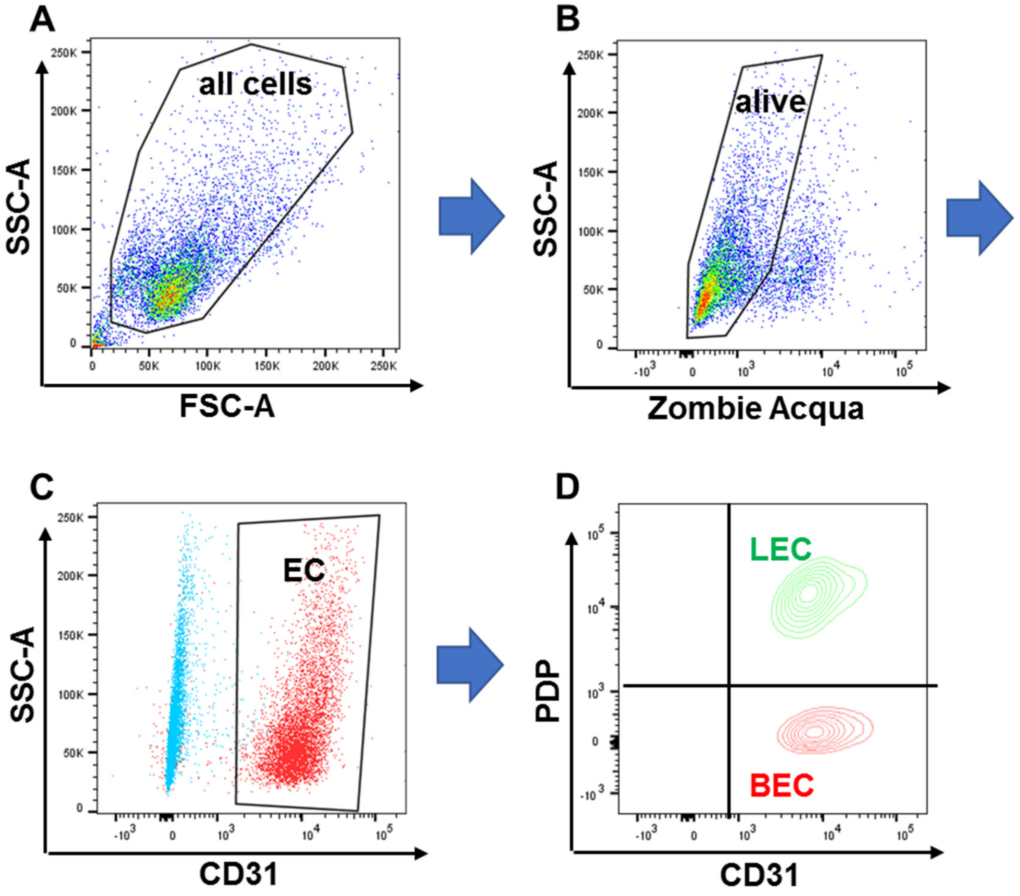

Figure S1. Gating strategy for flow cytometric analysis of HDMEC

Hierarchical gating strategy involved in the sequential exclusion of debris (**A**), followed by exclusion of dead cells (**A'**), and finally non-endothelial cells (**A''**). HDMECs were finally separated into LEC ($CD31^{+}PDP^{+}$) and BEC ($CD31^{+}PDP^{-}$) according to PDP expression (**A'''**).

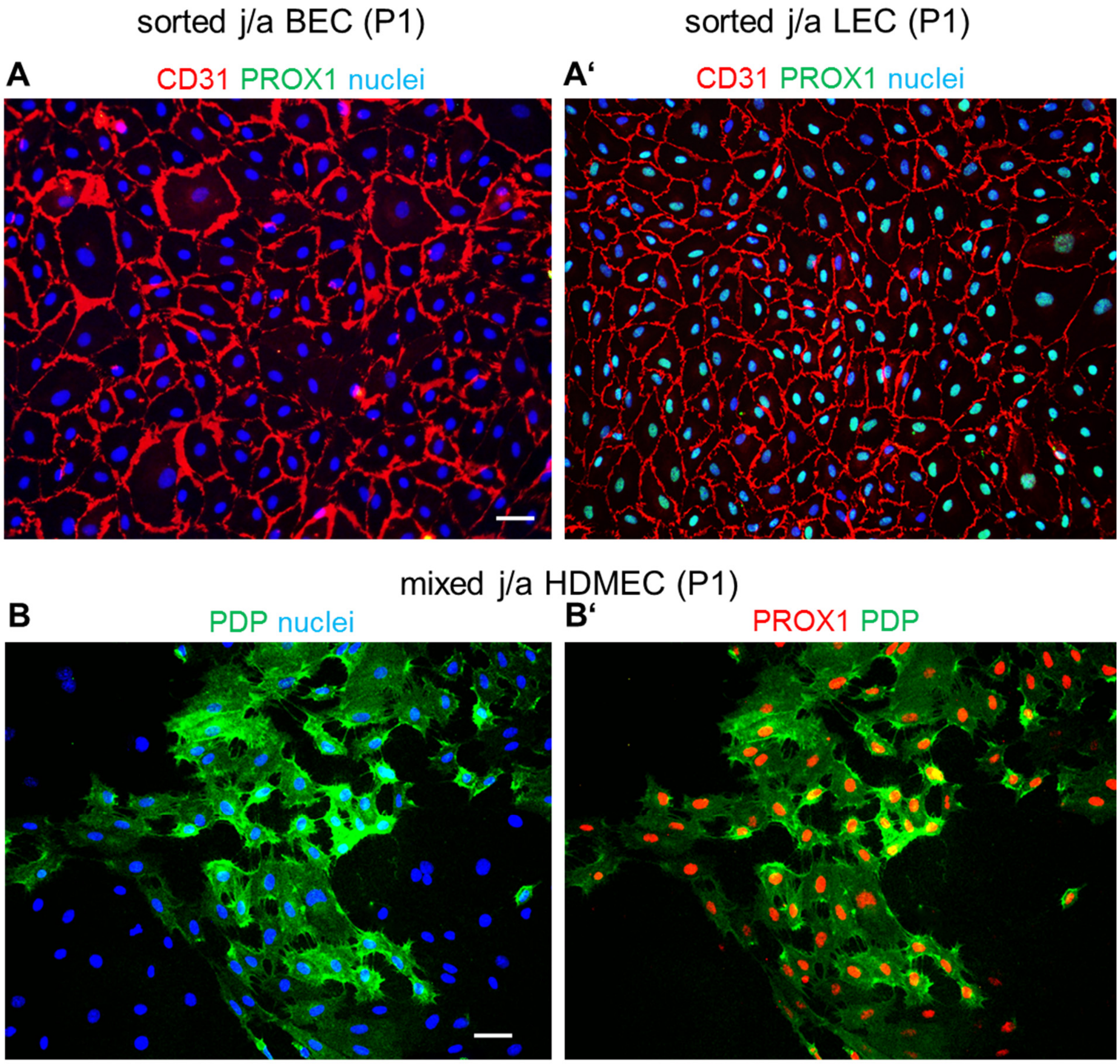

Figure S2. Purity of sorted j/a BEC and LEC

(**A**, **A'**) Purity of sorted j/a BEC and j/a LEC was confirmed at P1 using immunofluorescence strainings. The exclusive presence of CD31^{+}/Prox1^{-} endothelial cells indicated a pure BEC population (A), whereas a pure LEC population was confirmed by the sole presence of CD31^{+}/Prox1^{+} endothelial cells (A'). (**A**, **A'**) Mixed (unsorted) HDMEC showed that specifically and exclusively only podoplanin positive (PDP^{+}) LEC also Prox1^{+} expressed (n=3). Scale bars: 50 µm).

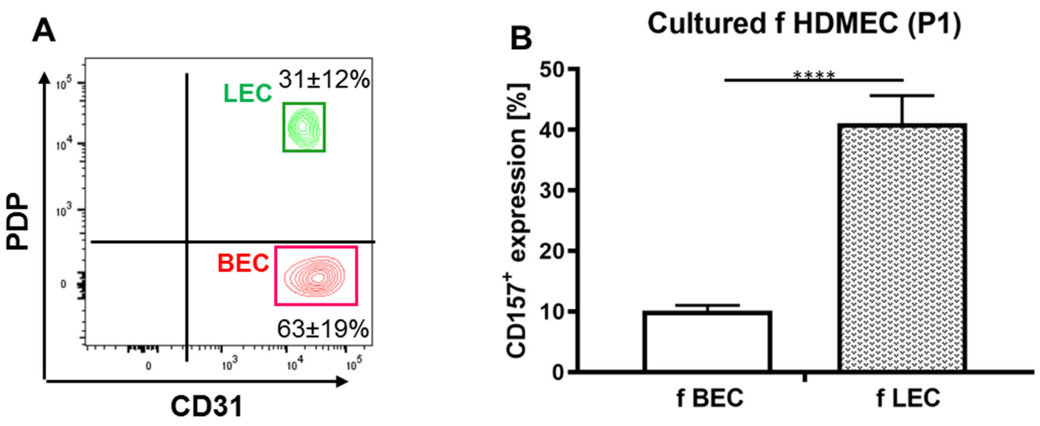

Figure S3. CD157 expression in cultured fetal HDMEC (P1)

(**A**) Previously cultured fetal HDMEC at P1 show a distinct separation into BEC (63±19%) and LEC (31±12%) (n=5). (**B**) CD157 expression on cultured fetal HDMEC at P1 is significantly higher in the fraction of fetal LEC (41.1±15.1%) than in fetal BEC (10.1±2.9%) ($p<0.0001$; n=5). Statistical tests were performed using unpaired student t-test.

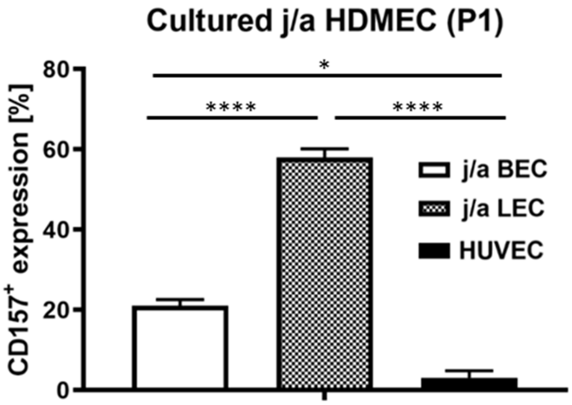

Figure S4. CD157 expression in cultured juvenile/adult HDMEC (P1)

Previously cultured and separated juvenile/adult HDMEC at P1 revealed that only 21.06±11.6% of dermal j/aBEC expressed CD157 marker, whereas 58.03±16.8% (p <0.000) of j/aLEC were CD157 positive a distinct separation into BEC (63±19%) and LEC (31±12%). Further, only a few HUVEC expressed CD157 (3.13±1.71%) (<0.0001 vs. j/aLEC and p=0.0226 vs j/aBEC) (n=20). Statistical tests were performed using unpaired student t-test.

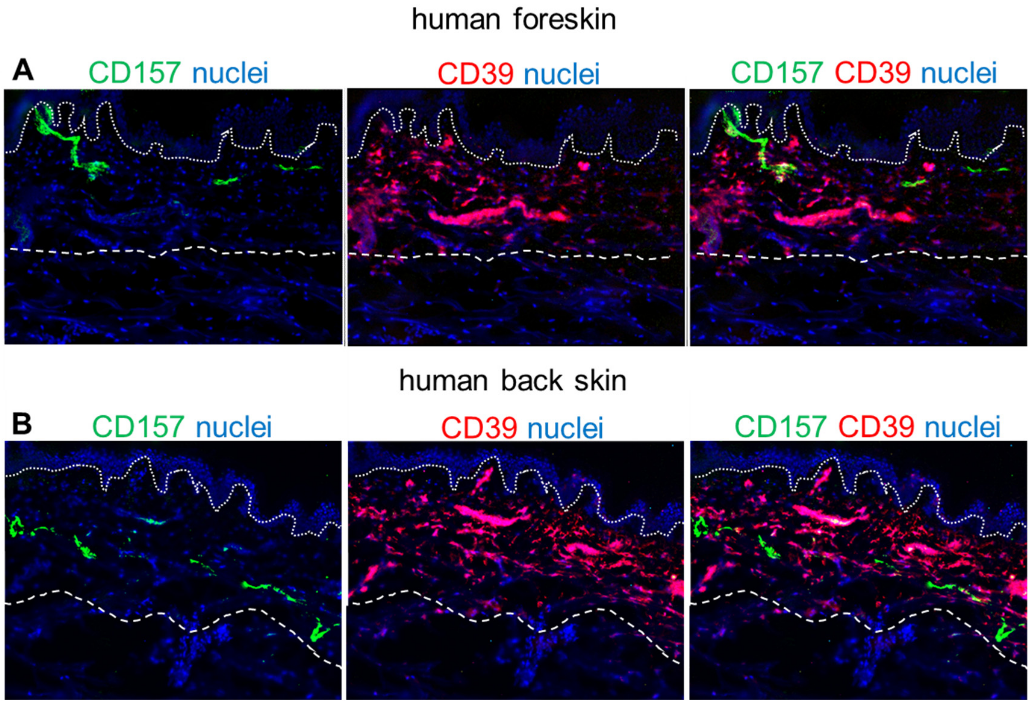

Figure S5. CD157 expression in papillary/reticular dermis of human skin

(A-B) Confocal immunofluorescence images of j/a foreskin (A) and back skin (B) stained for CD39 (red) and CD157 (green). Note that CD157^{+} capillaries are restricted to the CD39 positive papillary dermis, whereas they are absent in the lower reticular dermal part. Shown are single stainings as well as merged overlays. Dotted lines delineate the dermo-epidermal junction.

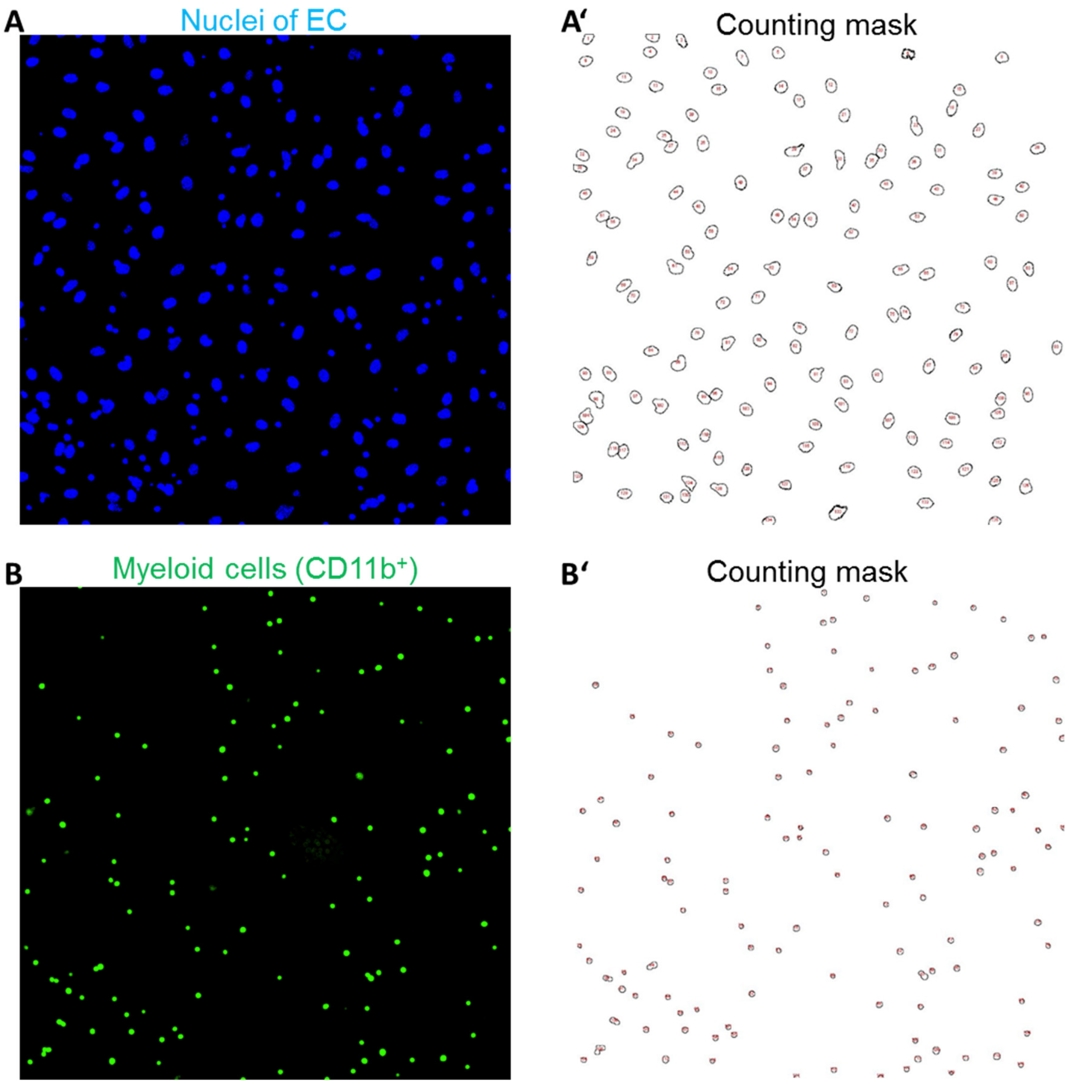

Figure S6. Quantification of immune binding of CD11b immune cells to CD157^{+} endothelial cells.

(A-B') Example for the quantification of adherent myeloid CD11b positive blood cells. CD157^{+} HDMECs were counterstained with Hoechst (**A**) and a counting mask showing the outlines was generated using a Fiji software (**A'**). CD11b^{+} myeloid cells were stained with a green cell tracker (**B**) and an equivalent counting mask was generated in Fiji (**B'**). As the cells were clearly distinguishable by size, the ratio of immune (myeloid) cells to HDMEC could be calculated.
